# Supplementary material for: Scanning transcriptomes for nonlinear, domain-level similarities using hmSEEKR
Source: bioRxiv. 2026 Jul 8:2026.07.03.736302. Preprint. [Version 1] doi: 10.64898/2026.07.03.736302 (PMC13370960; doi:10.64898/2026.07.03.736302)
Supplement: Supplement 1 [file NIHPP2026.07.03.736302v1-supplement-1.pdf]

bioRxiv preprint doi: https://doi.org/10.64898/2026.07.03.736302; this version posted July 8, 2026. The copyright holder for this preprint (which was not certified by peer review) is the author/funder, who has granted bioRxiv a license to display the preprint in perpetuity. It is made available under a CC-BY 4.0 International license.

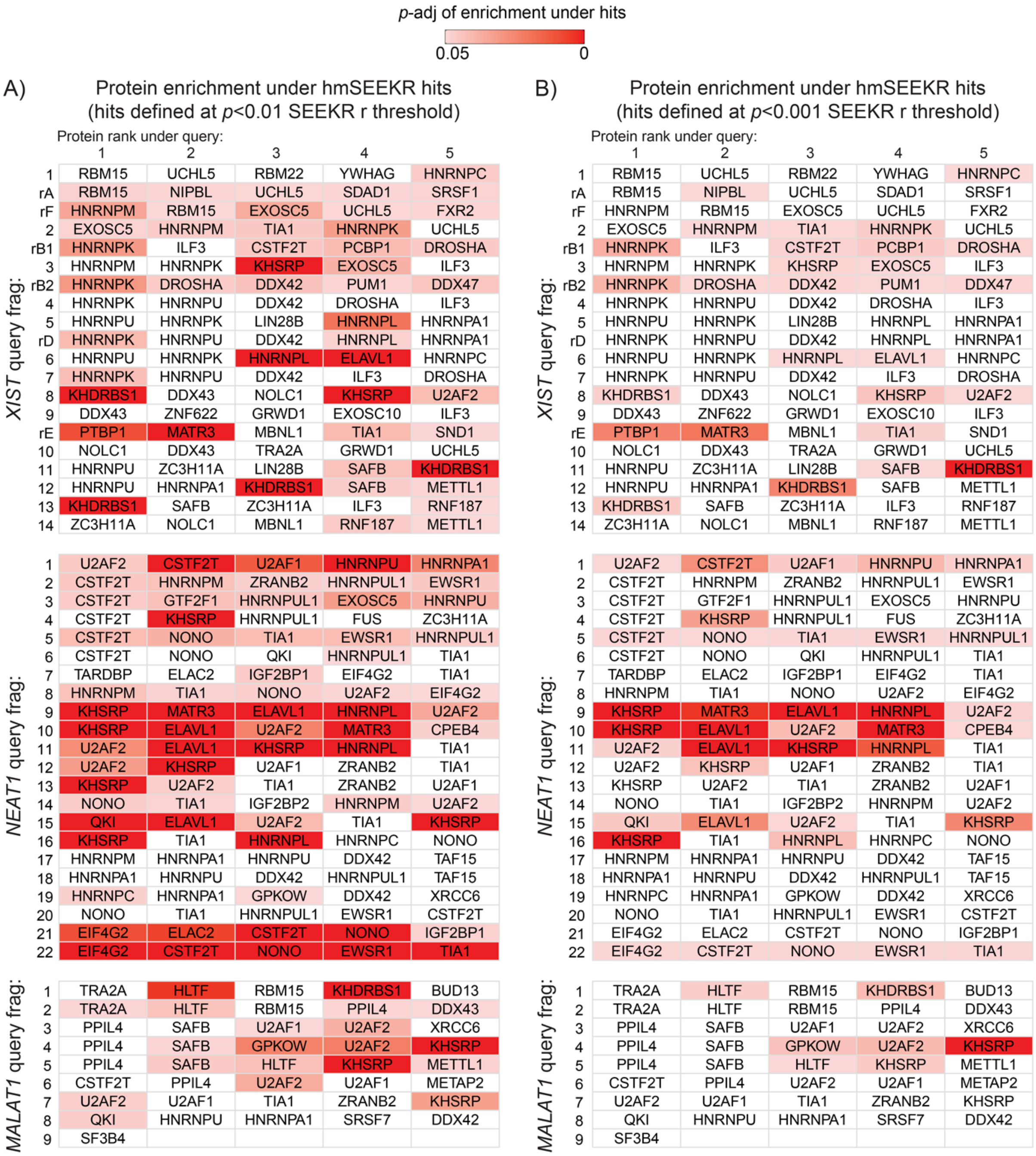

Figure S1. Protein enrichment under hits to query domains at *k*-mer length *k* = 6 and thresholds for hmSEEKR hit definition of *p* < 0.01 (A) and < 0.001 (B). Each row in (A) and (B) corresponds to a query domain and is displaying the Wilcoxon signed-rank test, Benjamini-Hochberg adjusted *p* value of CLIP enrichment for each of that query's top five most enriched proteins underneath the hits to the query. Related to Figure 3.

bioRxiv preprint doi: https://doi.org/10.64898/2026.07.03.736302; this version posted July 8, 2026. The copyright holder for this preprint (which was not certified by peer review) is the author/funder, who has granted bioRxiv a license to display the preprint in perpetuity. It is made available under a CC-BY 4.0 International license.

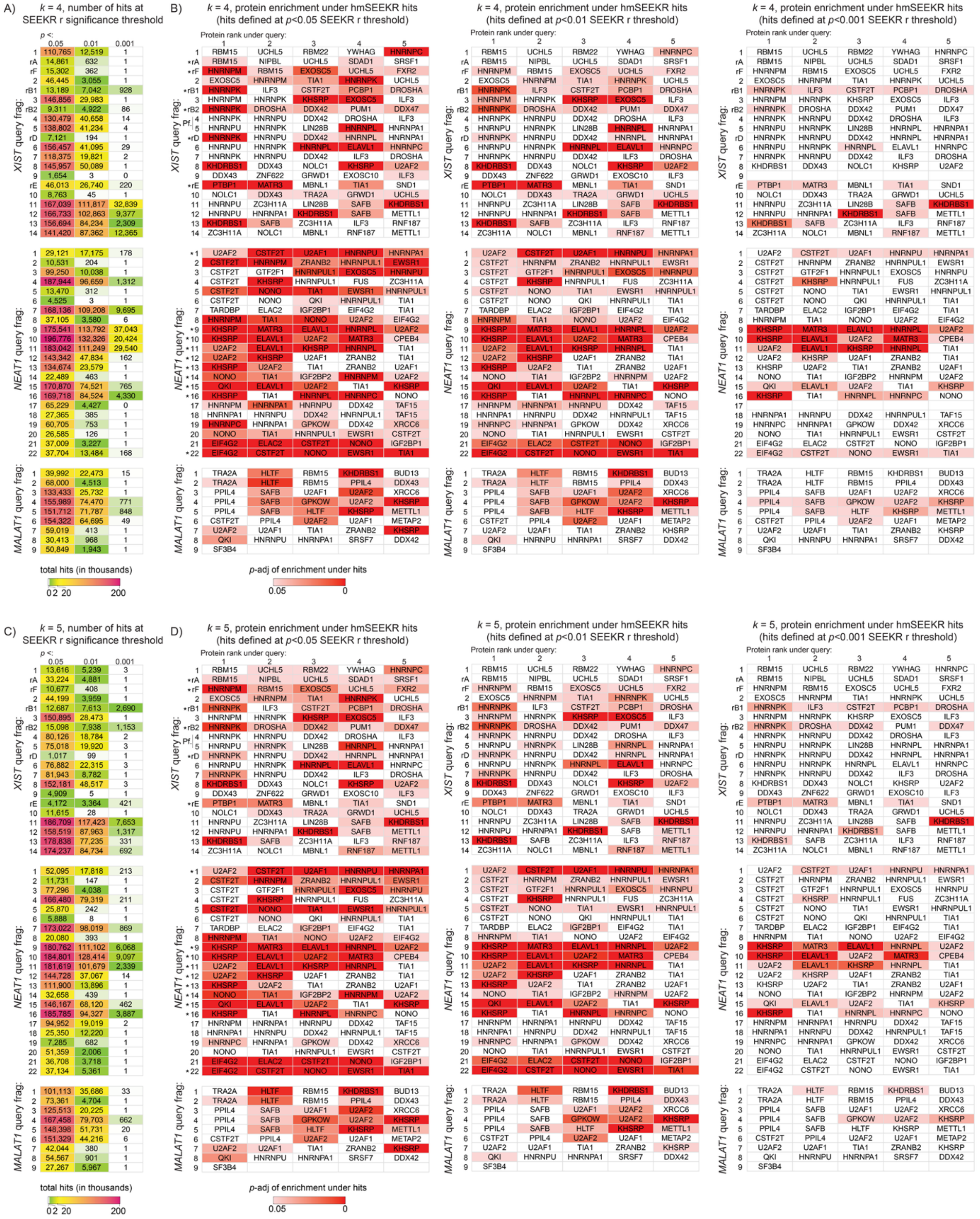

bioRxiv preprint doi: https://doi.org/10.64898/2026.07.03.736302; this version posted July 8, 2026. The copyright holder for this preprint (which was not certified by peer review) is the author/funder, who has granted bioRxiv a license to display the preprint in perpetuity. It is made available under a CC-BY 4.0 International license.

Figure S2. Location of hmSEEKR hits and enrichment of CLIP data under hits with searches performed at *k*-mer length *k* = 4 and 5. (A) At *k*-mer length *k* = 4, number of hits to each *XNM* query in the set of genes that produce chromatin-associated RNAs in K562 cells. **(B)** At *k*-mer length *k* = 4, significance of enrichment of CLIP signal under hmSEEKR hits versus shuffled controls for query-associated RBPs across all *XNM* query domains (Wilcoxon signed-rank test, Benjamini-Hochberg adjusted), at the $p < 0.05$, 0.01, and 0.001 thresholds for hit definition. **(C)** and **(D)**, same as (A) and (B), but for searches performed at *k*-mer length *k* = 5. Related to Figure 3.

bioRxiv preprint doi: https://doi.org/10.64898/2026.07.03.736302; this version posted July 8, 2026. The copyright holder for this preprint (which was not certified by peer review) is the author/funder, who has granted bioRxiv a license to display the preprint in perpetuity. It is made available under a CC-BY 4.0 International license.

A) *k* = 4, combinatorial enrichments relative to shuffled controls

query	RBP set	hits @ $p<0.05$	hits @ $p<0.01$	hits @ $p<0.001$
XrA	RBM15+SRSF1	0.3295	0.4156	0.5
XrE	PTBP1+MATRN3+TIA1	<2.2E-310	<2.2E-310	1.23E-55
M1	TRA2A+KHDRBS1	1.15E-229	3.49E-157	0.2414
M3	U2AF1+U2AF2	6.26E-19	6.84E-47	0.5
M6	U2AF1+U2AF2	0.6784	1.29E-10	0.0130

B) *k* = 5, combinatorial enrichments relative to shuffled controls

query	RBP set	hits @ $p<0.05$	hits @ $p<0.01$	hits @ $p<0.001$
XrA	RBM15+SRSF1	0.0723	0.9721	0.5
XrE	PTBP1+MATRN3+TIA1	9.28E-72	6.92E-68	1.76E-29
M1	TRA2A+KHDRBS1	<2.2E-310	1.12E-233	0.0524
M3	U2AF1+U2AF2	1.22E-64	3.65E-30	0.5
M6	U2AF1+U2AF2	1.92E-6	1.50E-20	0.9091

C) *k* = 6, combinatorial enrichments relative to shuffled controls

query	RBP set	hits @ $p<0.05$	hits @ $p<0.01$	hits @ $p<0.001$
XrA	RBM15+SRSF1	1.73E-40	2.41E-5	0.1388
XrE	PTBP1+MATRN3+TIA1	1.28E-140	1.52E-189	1.93E-89
M1	TRA2A+KHDRBS1	<2.2E-310	1.02E-251	2.73E-12
M3	U2AF1+U2AF2	5.99E-21	2.09E-13	0.6824
M6	U2AF1+U2AF2	0.0738	5.59E-4	0.5

Figure S3. Significance of combinatorial enrichments of CLIP signal under hmSEEKR hits for *XIST* and *MALAT1* query domains that have been previously shown to interact with more than one protein client. Significance of combinatorial enrichment (Fisher's exact) is shown at each of three separate *p* value thresholds for hmSEEKR hit definition. Data shown for searches performed using *k*-mer lengths *k* = 4 **(A)**, 5 **(B)**, and 6 **(C)**. Related to Figure 3.

bioRxiv preprint doi: https://doi.org/10.64898/2026.07.03.736302; this version posted July 8, 2026. The copyright holder for this preprint (which was not certified by peer review) is the author/funder, who has granted bioRxiv a license to display the preprint in perpetuity. It is made available under a CC-BY 4.0 International license.

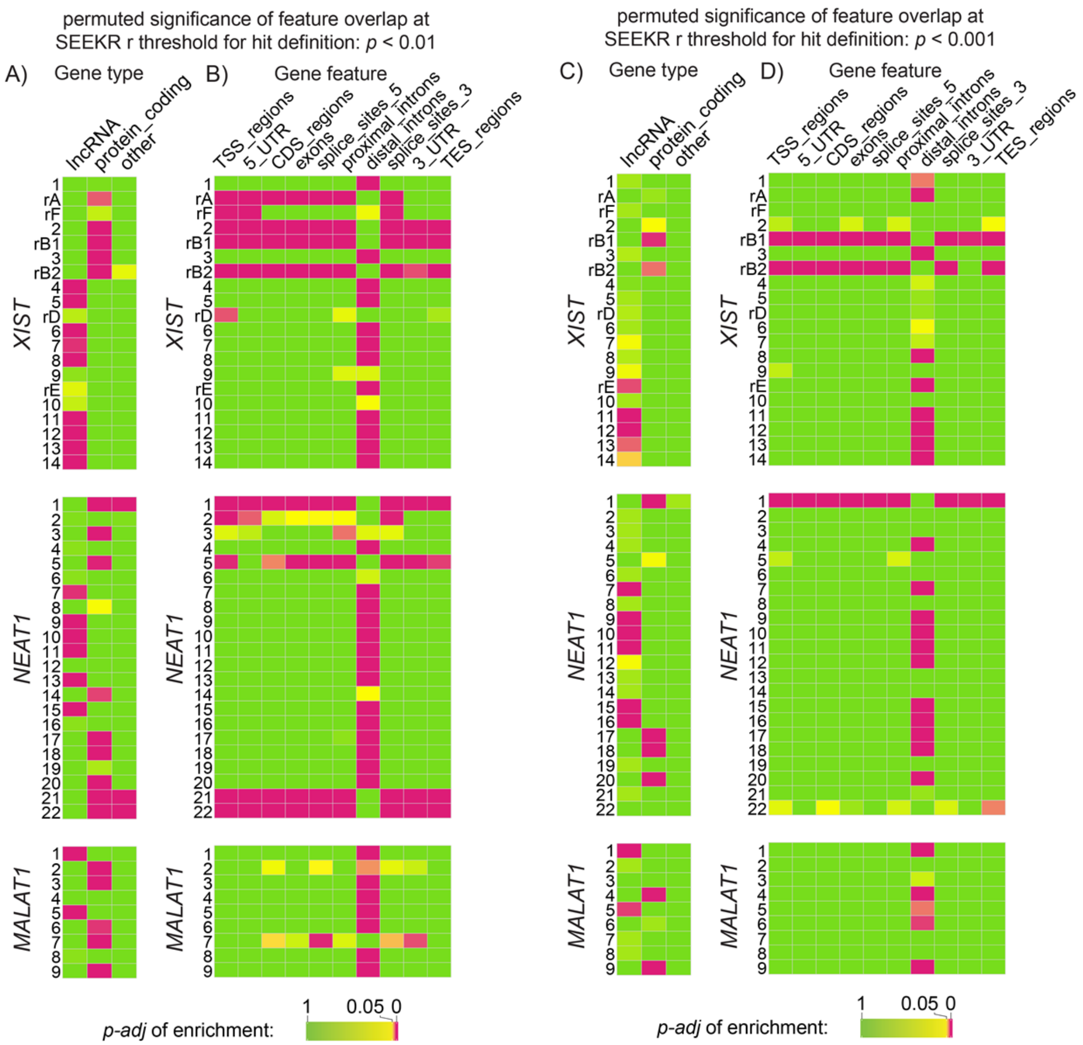

Figure S4. Gene-centric overlaps of hmSEEKR hits at *k*-mer length *k* = 6 and thresholds for hmSEEKR hit definition of $p < 0.01$ and $p < 0.001$. (A) For each *XNM* query, adjusted *p* value of hit enrichment in the set of genes that produce chromatin-associated RNAs in K562 cells (Fisher's exact, Benjamini-Hochberg adjusted)**;** and **(B)** *p* value of hit enrichment in the features of genes that produce chromatin-associated RNAs in K562 cells (Fisher's exact, Benjamini-Hochberg adjusted). Hits were defined using a SEEKR-derived *p* value threshold of $p < 0.01$. (C) and (D), same as (A) and (B) but using a SEEKR-derived *p* value threshold of $p < 0.001$. Related to Figure 4.
